# Elevated Anti-Müllerian Hormone Levels in Newborns of Women with Polycystic Ovary Syndrome: a Systematic Review and Meta-analysis Based on Observational Studies

**DOI:** 10.1007/s43032-021-00652-w

**Published:** 2021-06-15

**Authors:** Siyu Zhou, Danhua Lu, Shu Wen, Yongcheng Sheng, Deying Kang, Liangzhi Xu

**Affiliations:** 1 Department of Obstetrics and Gynaecology, West China Second University Hospital, Sichuan University, Chengdu, China; 2 Key Laboratory of Birth Defects and Related Diseases of Women and Children, Sichuan University, Ministry of Education, Chengdu, China; 3 Reproductive Endocrinology and Regulation Laboratory, West China Second University Hospital, Chengdu, Sichuan China; 4 West China School of Public Health and West China Fourth Hospital, Sichuan University, Chengdu, Sichuan China

**Keywords:** Polycystic ovary syndrome, Anti-Müllerian hormone, Metabolic health, Offspring

## Abstract

We performed this updated systematic review and meta-analysis to evaluate anti-Müllerian hormone levels (AMH) in newborns of mothers with polycystic ovary syndrome (PCOS) compared with healthy controls. A search of the literature was conducted in the PubMed, MEDLINE, EMBASE, Cochrane Library, CBM, CNKI, WANFANG, and VIP for articles to assess AMH levels in offspring of PCOS and non-PCOS mothers irrespective of language. These databases were searched from their inception to December 7, 2020. The quality of studies was assessed using the Newcastle-Ottawa Scale (NOS) scoring system. Standardized mean differences (SMDs) with 95% confidence intervals (CIs) were adopted to calculate the overall estimates with random-effects models. A total of 6 studies with 846 participants were included. The pooled analysis found an increased AMH level in the umbilical cord blood in newborns of PCOS mothers (SMD =0.62, 95% CI [0.28, 0.95]). Subgroup analyses revealed an elevation of AMH concentrations in female neonates, neonates born to American and Asian PCOS mothers. In addition, higher AMH levels were also found in studies diagnosed by the National Institute of Health (NIH) criteria, maternal clinical/biochemical hyperandrogenism, or maternal body mass index (BMI) >30 kg/m^2^. Meta-regression analysis suggested that diagnostic criterion contributed mostly to the high heterogeneity. We demonstrated that AMH levels in neonates born to PCOS mothers were essentially higher, which indicates that AMH may act as an enigmatic role in the pathogenesis of PCOS which inhibits folliculogenesis in the fetal stage.

Polycystic ovary syndrome (PCOS) is a common metabolic and endocrine disorder that affects about 8 to 13% females in their reproductive lifespan according to criteria used [1]. It is characterized by ovulatory dysfunction, androgen excess (primarily ovarian but also adrenal in origin), and the appearance of polycystic ovaries on ultrasound [2]. The etiology and pathogenesis of the syndrome are recognized as multifactorial and heterogeneous; previous studies demonstrated that the first-degree female relatives of individuals with PCOS were more often diagnosed with PCOS compared with non-PCOS daughters in their puberty and sexual maturity [3, 4]. Recent evidence was indicative of the disturbances of steroid (especially exposure to hyperandrogenism) during intrauterine life which had been implicated in the origin of PCOS and might modify the reproductive and metabolic function of offspring [5, 6].

Anti-Müllerian hormone (AMH) is a member of the transforming growth factor (TGFβ) superfamily and is clinically used as a marker for evaluating ovarian reserve [7]. As a reflection of the increased stock of the preantral and small antral follicles, serum AMH levels were found significantly 2–4 folds ascended in PCOS individuals and were positively associated with total testosterone levels and free androgen index levels [8, 9]. Previous studies reported that peripubertal daughters of PCOS women exhibited higher levels of AMH, validating that the follicular alterations that appeared in adult PCOS might happen early during development [10, 11]. Moreover, a slice of studies exhibited that pregnant women with PCOS had higher serum AMH levels during the early trimester and at delivery, compared with healthy controls [12, 13]. Experimental studies in monkeys, sheep, and rodent animals supported the hypothesis of exposure to excessive steroid, or AMH might develop PCOS-like phenotypes in the offspring [14, 15]. Quite a few research provided evidence of a critical window of susceptibility during differentiation of organs and systems that could “program” ovary in the female during fetus period and, subsequently, lead to PCOS [16, 17].

Although numerous animal studies laid the foundations of this developmental hypothesis for PCOS etiology [18, 19], however, this theory was doubted in human for many years, and insufficient observational human studies were conducted with controversial results. The latest meta-analysis concluded that fetal cord blood testosterone and dehydroepiandrosterone (DHEA) levels were not related to PCOS, while the androstenedione (ADION) levels were tended to be reduced in newborns of PCOS mothers [20], which was opposite to the present hypothesis. It could be explained that maternal testosterone might be bound by sex hormone-binding globulin (SHBG) and then quickly degraded and converted by the placenta’s high levels of aromatase into estradiol. This mechanism has greatly contributed to protecting the fetus from a hyperandrogenic state [21].

Furthermore, there was little evidence substantiating a relationship between the exposure to maternally derived androgens and subsequent alteration of ovarian functions during reproductive life [13, 22–26]. Only a few studies with limited sample size had previously compared cord blood characteristics of offspring of PCOS mothers, generating conflicting outcomes. Several investigations reported a higher AMH in the neonates born to PCOS mothers than to control mothers [23–26], while the others reported no difference between these two groups [13, 22]. By evaluating the AMH levels of newborns, the present meta-analysis and systematic review was constructed to find potential evidence of influence by the intrauterine environment. Our research was registered on PROSPERO under the number CRD42021231717.

## Materials and Methods

### Inclusion Criteria of Studies

Studies were included if they met all following criteria: (1) observational studies that were published in peer-reviewed journals irrespective of language; (2) studies reported data of pregnant individuals diagnosed as PCOS (according to the Rotterdam criteria [27] or the National Institutes of Health (NIH) criteria [28]); and (3) studies provided hormonal means and standard deviations (SDs) or sufficient data to calculate them. Studies were excluded if they (1) were letters, case reports, editorials, animal experiments, or conference abstracts; (2) included individuals of twin or multiple pregnancies; and (3) included individuals with ovarian surgery history, ovarian radiotherapy, systemic chemotherapy, severe pregnancy complications, or immune diseases.

### Search Strategies

A search of the literature to the end of to December 7, 2020, was conducted in the PubMed, MEDLINE, EMBASE, Cochrane Library, CBM, CNKI, WANFANG, and VIP for articles to assess AMH levels in newborns of mothers with PCOS in comparison with healthy controls. The search terms were included as follows: (polycystic ovary syndrome [Mesh] or PCOS) and (adult offspring [Mesh] or newborn or child of impaired parents [Mesh] or infant or fetus or neonate) and (AMH or anti-Müllerian hormone). The reference lists of retrieved publications and relevant reviews were manually searched to identify any missing relevant articles.

### Study Selection and Data Extraction

Two review authors (S.Z. and D.L.) independently screened the title and abstract and selected the articles. Full texts were retrieved for further assessment. The characteristics of included studies were extracted according to Cochrane guidelines by 2 authors (S.Z. and D.L.). Discrepancies were noted and resolved by discussing with the third author (L.X.).

### Quality Assessment

The quality of selected studies was assessed using the Newcastle-Ottawa Scale (NOS) scoring system [29]. According to the quality score assessment, the total score ranged from 0 to 9. Studies with a score of 7 or above were considered high-quality, and studies with a score of 4 or below were considered low-quality. Studies with a score between 4 and 7 were considered medium-quality. Evaluation of evidence quality (high-quality, medium-quality, or low-quality) was determined by 2 review authors (S.Z. and D.L.) independently, with differences resolved by the discussion (S.Z., D.L., and L.X.)

### Data Synthesis and Statistical Analysis

Hormone levels were described as mean ± standard deviation (SD) in most studies while extracted as median and range in one study and extracted as median and interquartile range in another study; means and SDs were estimated by the method provided by Wan et al [30] and Luo et al [31]. Standardized mean differences (SMDs) with 95% confidence intervals (CIs) were adopted to calculate the overall estimates. Heterogeneity across studies was quantified using the Q-statistic and inconsistency index (I^2^). When I^2^ > 50%, heterogeneity was considered severe; when 25% <I^2^ <50%, heterogeneity was considered moderate; and when I^2^ < 25%, heterogeneity was considered low. In case of severe heterogeneity, a random-effects model was used.

Due to the fact that results revealed statistically notable heterogeneity, subgroup analyses were performed where available to investigate the potential source of inconsistencies (e.g., gender, geographical region, diagnostic criterion, measure method, birth weight, maternal androgen, maternal fasting insulin, and maternal body mass index (BMI)). Birth weight was categorized into two groups: a normal birth weight (NBW) group (birth weight <4000g) and a macrosomia group (birth weight >4000g). Maternal androgen was categorized into two groups: a hyperandrogenic group which presented clinical/biochemical hyperandrogenism and a normal androgenic group which presented no above characteristics. The cutoff value of maternal BMI was 30 kg/m^2^ according to the World Health Organization (WHO) criteria [32] as most studies included in our meta-analysis were based in the Americas and Europe.

When considerable heterogeneity was still presented after subgroup analyses, meta-regression analysis was conducted to further investigate the source of heterogeneity. Funnel plots of the outcomes enrolled the most studies to detect publication bias, and Egger’s test was also used to assess the publication bias of selected studies. Sensitivity analysis was performed where appropriate to determine the robustness of the results. Two-sided p < 0.05 was considered to be of statistically significance. All analyses were carried out in Review Manager software (Version 5.3; Copenhagen: The Nordic Cochrane Centre, The Cochrane Collaboration, 2014) and STATA software (Version 13.0; Stata Corporation, College Station, TX).

## Results

### Study Selection

**Fig. 1:**
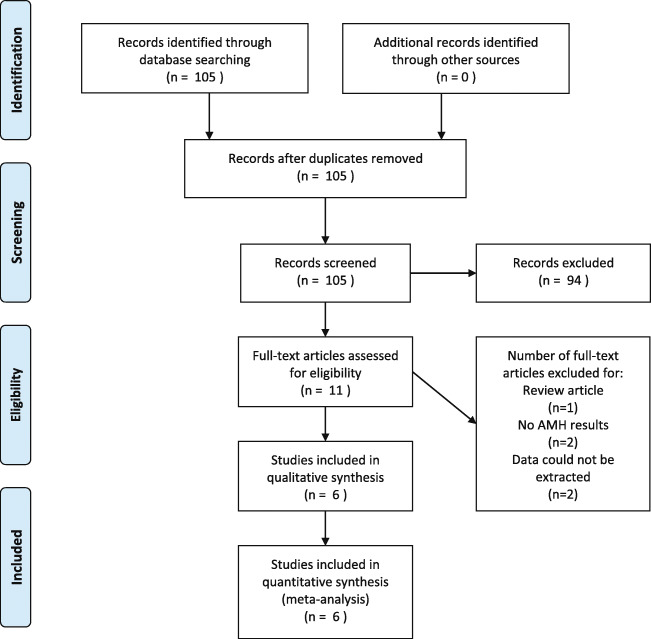
PRISMA flow diagram

A total of 105 citations were identified in electronic databases after removing duplications. Of these, 94 studies were excluded after screening by title and abstract. Five studies were excluded for reasons after screening the full texts: 1 study was a review, 2 studies reported no AMH outcomes, and 2 studies were lack of extractable data. Finally, 6 papers including 3 case-control studies, 2 prospective cohort studies, and 1 cross-sectional study were included in this review [13, 22–26]. The selection process was documented with a flowchart of Preferred Reporting Items for Systematic Reviews and Meta-Analyses (Fig. 1).

### Characteristics of Included Studies

The details of the included clinical studies were listed in Table 1. A total of 292 newborns of PCOS mothers and 574 newborns of healthy controls were reported. Three studies [23–25] were conducted in the Americas, 2 studies [13, 22] were conducted in Europe, and 1 study [26] was based in Asia. Most studies reported maternal testosterone levels, while only two studies reported maternal fasting insulin levels [23, 25]. Since most studies obtained the samples from mixed umbilical cord blood, results of Detti et al [24] from umbilical arterial and venous cord blood separately were synthesized. Most studies reported AMH levels in female and male offspring separately except for one study, which did not mention the gender of newborns [23]. Mean age of mothers and gestational age at birth between the PCOS group and control group were comparable in all studies as reported.

**Table 1:** Characteristics of included studies

First author	Year	Country	Study design	Diagnostic criterion	Mean age of mothers (years)	BMI of mothers	Gestational age at birth (days)	Measurement method for AMH	Sample size		Main outcomes
									PCOS	Control	
Caanen	2016	Britain	Case-control study	The Rotterdam criteria	33.40±3.96	24.93±3.92	277.39±2.35	ELISA	20	83	T,ADION,DHEA,E1,E2,E3,AMH
Crisosto	2012	Chile	Case-control study	The NIH criteria	28±13.64	30.49±9.29	270.22±9.96	EIA	23	35	AMH,LH,FSH,17-OHP,A,T,E2
Detti	2019	America	Cross-sectional study	The NIH criteria	25.16±6.19	33.75±6.05	273.32±9.01	ECLIA	36	21	AMH,T,FSH,E2
Kollmann	2019	Austria	Prospective cohort study	The Rotterdam criteria	30.35±5.01	29.06±5.22	280.80±7.50	ELISA	79	354	T,fT,AMH,ADION
Sir-Petermann	2006	Chile	Case-control study	The NIH criteria	26.46±5.89	27.47±5.36	Not mentioned	EIA	14	21	GnRH,T,ADION,E2,17-OHP,SHBG,AMH,inhibin B
Tadaion Far	2019	Iran	Prospective cohort study	The NIH criteria	27.71±5.17	Not mentioned	264.60±3.62	ELISA	120	60	AMH

*BMI* body mass index, *AMH* anti-Müllerian hormone, *PCOS* polycystic ovary syndrome, *T* testosterone, *ADION* androstenedione, *DHEA* dehydroepiandrosterone, *E1* esterone, *E2* estradiol, *E3* estriol, *LH* luteinizing hormone, *FSH* follicle-stimulating hormone, *17-OHP* 17*-*hydroxyprogesterone, *A* androstenedione, *NIH* National Institute of Health, *fT* free testosterone, *GnRH* gonadotropin-releasing hormone, *SHBG* sex hormone binding globulin, *ELISA* enzyme-linked immunosorbent assay, *EIA* enzyme immunoassay, *ECLIA* electro-chemiluminescence assay

### Methodological Quality Assessment of Included Studies

The Newcastle-Ottawa Scale (NOS) was used to fulfill methodological quality assessment (Table 2). The scores ranged from 7 to 9 stars, and all studies were considered high-quality. Nevertheless, some limitations can be found in some researches. First, the history of hospital controls was not elaborated in one study [13]. Secondly, there was no reliable medical of patients provided in one prospective cohort study [26], in which the control group was recruited from another resource of population that is different from the case group. In addition, another observational study [24] recruited hospital controls instead of community controls while a reliable exposure record was absent. One thing worth being mentioned is that all studies provided superior comparability of cases and controls on the basis of the design and analysis. As the hormone levels were measured in neonates with PCOS mothers and healthy mothers, no data was missing, and the non-response rates were considered 0.

**Table 2:** Methodological quality assessment of included studies

First author	Year	selection				Comparability of cases and controls on the basis of the design or analysis^a^	Exposure			Total score
		Adequate definition of the cases	Representativeness of the cases	Selection 2of controls	Definition of controls		Ascertainment of exposure	Same method of ascertainment for cases and controls	Non-response rate	
Caanen	2016	★	★	★	★	★★	★	★	★	9
Crisosto	2012	★	★	★	★	★★	★	★	★	9
Detti	2019	★	★		★	★★		★	★	7
Kollmann	2019	★	★	★		★★	★	★	★	8
Sir-Petermann	2006	★	★	★	★	★★	★	★	★	9
Tadaion- Far	2019	★			★	★★	★	★	★	7

^a^A maximum of 2 stars can be allotted in this category, one for age, BMI, and gestational age at labor and the other for other controlled factors

### Association of Maternal PCOS and AMH Levels in Neonates

**Fig. 2:**
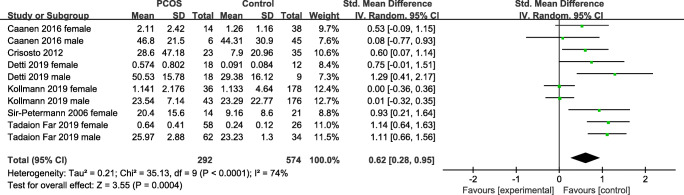
Forest plot

All 6 observational studies reported AMH levels in neonates. A meta-analysis based on the random-effects model of these 6 studies showed a noteworthy rise of the AMH level in neonates of PCOS mothers compared with control groups of healthy mothers, with an overall SMD =0.62, 95% CI (0.28, 0.95), Q test p-value <0.0001, and I^2^ = 74%, suggesting heterogeneity among these studies (Fig. 2).

The subgroup analyses were conducted based on fatal gender, geographical region, diagnostic criterion, measure method, birth weight, maternal androgen, and maternal BMI (Table 3). Pooled analyses demonstrated higher AMH levels in female offspring (SMD = 0.64; 95% CI = [0.14, 1.14]). Increased AMH levels were also observed in offspring of American (SMD = 0.81; 95% CI = [0.47, 1.16]) and Asian (SMD = 1.12; 95% CI = [0.79, 1.46]), PCOS mothers diagnosed by the NIH criteria (SMD =0.97; 95%CI = [0.74, 1.21]), hyperandrogenistic mothers (SMD =0.66; 95% CI = [0.205, 1.114]), and maternal BMI > 30 kg/m^2^ (SMD =0.78; 95% CI = [0.39, 1.17]), irrespective of measurement method or birth weight. However, no statistically notable discrepancy was observed in AMH levels between PCOS mothers’ neonates and healthy mothers’ neonates in the subgroup of male neonates (SMD =0.60; 95% CI = [−0.11, 1.31]), groups of European mothers (SMD =0.08; 95% CI = [−0.14, 0.30]), diagnosed by the Rotterdam criteria (SMD =0.08; 95% CI = [−0.14, 0.30]), and with a maternal BMI <30 kg/m^2^ (SMD =0.22; 95% CI = [−0.09, 0.54]).

**Table 3:** Meta-analyses of subgroups

Subgroups	Number of study	Result		Heterogeneity	
		SMD (95% CI)	p	I^2^ (%)	p value
Gender					
Female	5	0.64 (0.14,1.14)	0.01	74	0.004
male	4	0.60 (−0.11,1.31)	0.10	84	<0.0002
Region					
Americas	4	0.81 (0.47, 1.16)	<0.0001	0	0.61
Europe	4	0.08 (−0.14, 0.30)	0.49	0	0.51
Asia	2	1.12 (0.79, 1.46)	<0.0001	0	0.94
Diagnostic criteria					
Rotterdam	4	0.08 (−0.14, 0.30)	0.49	0	0.51
NIH	6	0.97 (0.74, 1.21)	<0.0001	0	0.63
Maternal androgen					
High	4	0.66 (0.205, 1.114)	0.004	29	0.238
Normal	4	0.30 (−0.087, 0.695)	0.127	66	0.031
Method					
ELISA	7	0.54 (0.11, 0.96)	0.01	81	<0.0001
Other	3	0.78 (0.39,1.17)	0.0001	0	0.43
Birth weight					
Macrosomia	2	0.72 (0.29, 1.15)	0.001	0	0.48
NBW	4	0.64 (0.20, 1.08)	0.004	24	0.27
Maternal BMI					
<30	5	0.22 (−0.09, 0.54)	0.16	45	0.12
>30	3	0.78 (0.39, 1.17)	0.0001	0	0.43

*SMD* standardized mean difference, *CI* confidence interval, *p* p values of SMD, *I*^*2*^ the value of I-squared statistics, *p value* p values of heterogeneity chi-squared, *NIH* National Institutes of Health, *ELISA* enzyme-linked immune-sorbent assay, *NBW* normal birth weight, *BMI* body mass index

### Meta-regression

Meta-regression was conducted to investigate potential heterogeneity sources. Gender, geographical region, diagnostic criterion, measure method, birth weight, maternal androgen, and maternal BMI were used as predefined factors (Table 4). Diagnostic criterion contributed crucially to the heterogeneity (p=0.001). The REML estimate of between-study variance decreased from 0.21 to 0 when we put diagnostic criterion into univariate meta-regression.

**Table 4:** Univariate meta-regression analysis for potential variables between studies

	Studies	I^2^ res (%)	Adjusted R^2^ (%)	Coefficient	**SE**	t	p	95% CI
Birth weight	6	16.02	0	0.083	0.319	0.26	0.808	(−0.802, 0.968)
Infant gender	9	80.47	−17.68	0.049	0.394	0.12	0.905	(−0.883, 0.980)
Maternal androgen	8	54.10	17.17	−0.358	0.333	−1.08	0.323	(−1.172, 0.455)
Diagnostic criteria	10	0	100.00	−0.910	0.166	−5.50	0.001	(−1.292, −0.528)
Maternal BMI	8	36.69	60.02	−0.602	0.292	−2.06	0.085	(−1.317, 0.113)
Method	9	71.85	−12.37	−0.205	0.375	−0.55	0.602	(−1.090, 0.681)
Study region	10	76.65	−13.73	0.044	0.230	0.19	0.855	(−0.488, 0.575)

*I*^*2*^ *res* the value of I-squared statistics of residual error, *R*^*2*^ the value of R-squared statistics, *SE* standard error, *t* t value of t test, *p* p value of t test, *CI* confidence interval, *BMI* body mass index

### Sensitivity Analysis and Publication Bias

**Fig. 3:**
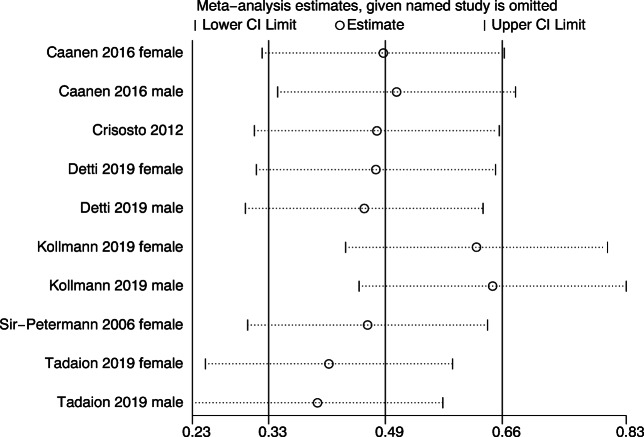
Sensitivity analysis of the included studies

**Fig. 4:**
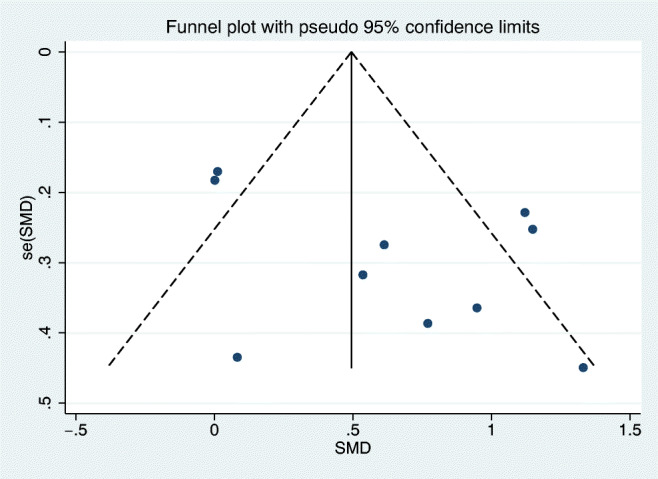
Funnel plot of the included studies

**Fig. 5:**
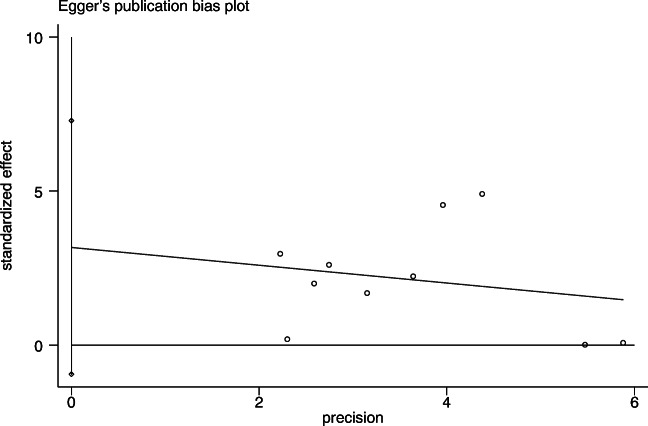
Egger’s publication bias plot

Sensitivity analysis showed that the study of Kollmann et al [13] made a remarkable contribution to the high heterogeneity (Fig. 3). By comprehensively scanning the research, an unmatched excessive number of control group and the wide range of AMH concentration might lead to the discrepancy. After excluding the study, I^2^ decreased from 74 to 19% with a constant result (SMD =0.85; 95% CI = [0.60, 1.09]). The funnel plot of the outcomes was constructed to detect the possibility of publication bias (Fig. 4); upon visual inspection, funnel plot was not relatively symmetric and suggested a little risk of publication bias. Considering that the number of articles included is limited, Egger’s test was performed to assess the publication bias (Fig. 5). The p-value for the Egger’s test was p=0.553, suggesting no potential publication bias in involved studies.

## Discussion

Our results demonstrated that, compared with neonates of healthy mothers, AMH levels significantly grown in neonates of PCOS mothers. After deleting Kollmann’s research, the I^2^ decreased to 19% while results remained stable. Subgroup analyses according to predefined factors including neonatal gender, geographical region, measurement method, birth weight, diagnostic criterion, maternal androgen, and maternal BMI suggested that higher AMH levels were observed female neonates and neonates born to American and Asian PCOS patients, diagnosed by the NIH criteria, with maternal hyperandrogenism or maternal BMI >30 kg/m^2^. This meta-analysis supported the hypothesis that maternal intrauterine environment disorders the ovarian function of the fetus and contributes to the elevation of AMH levels in umbilical cord blood, which promotes the development of PCOS during infancy. To our knowledge, this is the first meta-analysis evaluating the connection between maternal PCOS and AMH concentrations in neonates.

Currently, prenatal excess androgen exposure was shown to formulate alterations in AMH expression in preantral and antral follicles and at least in part mediate abnormal folliculogenesis [33]. Regarding AMH concentrations, controversy existed between observational studies for a long time. Our study synthesized all of these results and resolved the controversy by drawing a conclusion that increased AMH levels which were positively associated with maternal PCOS condition and maternal hyperandrogenism.

As generally considered, umbilical cord blood characteristics reflected maternal, placental, and fetal conditions, which might therefore represent potential disturbance of the intrauterine environment. In our meta-analysis, only two eligible studies reported maternal AMH levels [13, 24]. Interestingly, although higher AMH levels were revealed in PCOS mothers compared with healthy mothers in these two studies, there was no statistical discrepancy in AMH concentrations between female offspring of PCOS and non-PCOS mothers, which suggested that the AMH concentrations in umbilical cord blood might differ from that in maternal serum. Since peptide hormones were virtually unable to pass the placenta to a certain extent, it can be assumed that no passage of AMH existed from fetus to mother, similar to what occurs with insulin. Limited to maternal AMH data in included studies, the result should be treated with caution. It was universally acknowledged that higher AMH concentrations were in accordance with increased serum androgen [34]. It could be a possible mechanism in PCOS pathogenesis that AMH methylation or altered steroid receptor balance in the fetal ovary (granulosa cells) due to rising androgens results inhibition of follicle growth [35]. Reduced P450 aromatase and augmented androgen-producing enzyme activity were found in the placental tissue of women with PCOS, which impaired the placental protective function of the fetus from maternal hyperandrogenism and allowed the passage of testosterone through the placenta [36]. Retrospective researches expected that maternal testosterone might act on the fetal ovaries and recruited more preantral follicles to produce higher AMH levels when they become functional at around 36 weeks of gestation. The unbalance of steroid hormone in PCOS mothers also seemed to promote luteinizing hormone (LH) release and inhibit follicle-stimulating hormone (FSH) action on aromatase, adding to the hyperandrogenic environment of adult PCOS patients [37].

Another intrauterine environmental factor that might contribute to the elevation of AMH concentrations was increased maternal fasting insulin accompanied with elevated BMI [38]. In our meta-analysis, maternal fasting insulin levels were only detected in two studies, nevertheless, a consistent result that mothers with higher fasting insulin were more likely to give birth to neonates with an increase of AMH levels [23, 25]. This could be explained due to the abnormal effect of insulin action on AMH secretion by granulosa cells as suggested by Park et al in characters without PCOS [39]. Genetic factors might also underlie AMH overexpression in PCOS. Kevenaar et al. conducted a study by a genetic approach, which investigated the role of ALK2, a type I receptor for AMH/BMP signaling. It was also found that genetic variation within ACVR1 was associated with AMH levels and follicle numbers in PCOS women, suggesting that ALK2 signaling contributed to the disturbed folliculogenesis in PCOS patients [40]. These factors might be potential explanations for the correlation between maternal PCOS condition and elevated AMH levels in umbilical cord blood independent of excessive androgen, and it could be hypothesized that AMH methylation might represent another epigenetic alteration, related to a disturbed environment in utero in PCOS patients.

According to our meta-regression result, the diagnostic criterion was the main source that contributed to the high heterogeneity. The NIH criteria that were proposed in 1992 at a National Institute of Health sponsored conference on PCOS and revised in 2012 [41]. All studies included in this meta-analysis used the NIH criteria referred to the version of 1992 in which both clinical/biochemical hyperandrogenism and chronic anovulation were required for the diagnosis. A consensus workshop group sponsored by the European Society of Human Reproduction and Embryology and the American Society for Reproductive Medicine (ESHRE/ASRM) proposed a new criteria called the Rotterdam criteria in which at least two of the following three criteria were mandatory: oligo-anovulation, clinical/biochemical hyperandrogenism, and polycystic ovarian morphology (PCOM) on ultrasonography [27]. It was reported that overall prevalence rates of PCOS according to the Rotterdam criteria (with multiple sub-phenotypes) were twice as high as those according to NIH criteria in a recent meta-analysis [1]. Different results caused by these two criteria in our subgroup study also supported the consensus that the Rotterdam criteria were more comprehensive while the NIH criteria were more strict and specific. In addition, inconsistencies might also derive from sample types and measurement methods as shown in subgroup analyses.

As expected, AMH concentrations were notably higher in the umbilical cord blood of males than in females. However, the levels in PCOS boys and non-PCOS boys were similar. AMH had been proved effective during the period of sexual differentiation. In the male fetus, AMH was secreted by Sertoli cells of the testes from early fetal life, which remained a high level and thus induced physiological involution of the paramesonephric Müllerian ducts. Although the regression of the paramesonephric Müllerian ducts was complicated early in gestation, the production process of AMH by Sertoli cells continued until adulthood [42]. In the female fetus, the AMH was secreted by granulosa cells of the ovarian follicles from 36 weeks of gestation until menopause, whose expression peak was in preantral follicles and small antral follicles. However, the AMH was hardly produced in primordial follicles [37]. It could partially illustrate the difference in AMH concentrations between boys and girls. Furthermore, maternal PCOS state accompanied with hyperandrogenism or high AMH might have a more critical influence on ovaries than the testes in offspring; thus, more studies were required to verify the hypothesis.

There were quite a few superiorities in our systematic review and meta-analysis. First, all eligible studies were of high methodological quality according to the NOS scoring system, which might enhance the statistical power. Second, the result of sensitivity analysis confirmed the stability of observed differences in AMH levels in neonates of PCOS patients compared with controls. Finally, the result of Egger’s test indicated no obvious publication bias was detected.

However, important limitations must be carefully considered in our study. First, there was high heterogeneity between included studies; although we analyzed potential source of heterogeneity by sensitive analysis, subgroup analyses, and meta-regression analysis, there might be some other factors not considered which would affect the reliability of our conclusion. Second, the number of studies included was limited, and results from a small sample size should be understood with caution. Third, some missing and unpublished data might produce a certain degree of systemic bias and hospital-based case-control studies which might be susceptible to selection bias. Future researches with a large sample size investigating maternal PCOS status’ influence on the fetus are needed.

## Conclusion

In summary, the positive findings of this study suggested that AMH levels in neonates born to PCOS mothers were noticeably higher than those in neonates born to healthy controls. At present, AMH is a reliable biomarker of ovarian reserve and also clinically used to assist in the diagnosis of PCOS. We demonstrated that AMH might play a critical part in the PCOS pathogenesis which inhibits folliculogenesis in the fetal stage. The conclusion we draw from the pooled analysis may bring favorable repercussions to female reproductive health and propel the study of pathogenesis and etiology of PCOS.
